# Freedom within a cage: how patriarchal gender norms limit women’s use of mobile phones in rural central India

**DOI:** 10.1136/bmjgh-2021-005596

**Published:** 2021-09-22

**Authors:** Kerry Scott, Aashaka Shinde, Osama Ummer, Shalini Yadav, Manjula Sharma, Nikita Purty, Anushree Jairath, Sara Chamberlain, Amnesty Elizabeth LeFevre

**Affiliations:** 1 Department of International Health, Johns Hopkins University Bloomberg School of Public Health, Baltimore, Maryland, USA; 2 BBC Media Action, New Delhi, Delhi, India; 3 Oxford Policy Management, New Delhi, Delhi, India; 4 Independent researcher, New Delhi, India; 5 School of Public Health and Family Medicine, University of Cape Town, Cape Town, Western Cape, South Africa

**Keywords:** health education and promotion, health policy, maternal health, public Health, qualitative study

## Abstract

**Introduction:**

India has one of the highest gender gaps in mobile phone access in the world. As employment opportunities, health messaging (mHealth), access to government entitlements, banking, civic participation and social engagement increasingly take place in the digital sphere, this gender gap risks further exacerbating women’s disadvantage in Indian society. This study identifies the factors driving women’s unequal use of phones in rural Madhya Pradesh, India.

**Methods:**

We interviewed mothers of 1-year-old children (n=29) who reported that they had at least some access to a mobile phone. Whenever possible, we also spoke to their husbands (n=23) and extended family members (n=34) through interviews or family group discussions about the use of phones in their households, as well as their perspectives on gender and phone use more broadly. Our analysis involved comparing wife–husband pairs to assess differences in phone access and use, and thematic coding on the determinants of women’s phone use using an iteratively developed conceptual framework.

**Results:**

While respondents reported that women could use the phone without needing permission, this apparent ‘freedom’ existed in a context that severely constrained women’s actual use, most directly through: (1) narrow expectations and desires around how women would use phones, (2) women’s dependence on men for phone ownership and lower proximity to phones, (3) the poorer functionality of women’s phones; (4) women’s limited digital skills, and (5) time allocation constraints, wherein women had less leisure time and were subject to social norms that discouraged using a phone for leisure.

**Conclusion:**

Our framework, presenting the distal and proximate determinants of women’s phone use, enables more nuanced understanding of India’s digital divide. Addressing these determinants is vital to shift from re-entrenching unequal gender relations to transforming them through digital technology.

**Key questions**

**What is already known?**

- In most regions of the globe, women lag behind men in the use of digital technology; this gender gap is particularly wide in rural India.
- Mobile phone use can bring enormous benefits and is increasingly a prerequisite to accessing a wide range of health, education, social and financial benefits.
- The gender gap in phone use risks further entrenching inequality.

**What are the new findings?**

- Phone use among young married women in rural Madhya Pradesh was rarely constrained by overt gatekeeping, but was tightly constrained by proximate and distal barriers driven by patriarchal gender norms.
- Women’s phone use was limited by the narrow range of socially acceptable uses for women (speaking to family) compared with men (work, entertainment and socialising), women’s dependence on men for phone ownership and lower proximity to phones, the poorer functionality of women’s phones; women’s limited digital skills and time allocation constraints.

**What do the new findings imply?**

- Increasing reliance on mobile phones in social policy and programming risks exacerbating gender inequity.
- Gender transformative programming demands ongoing investment in mobile-based services for women designed to overcome multiple barriers identified—as well as non-mobile services for women without access to phones at all.

## Introduction

India has one of the largest gender gaps in mobile phone access in the world. Across all low-income and middle-income countries (LMICs), women are 8% less likely than men to own a phone (ie, 82% of women vs 89% of men own phones).^1^ However, in India, women are 20% less likely (ie, only 63% of women vs 79% of men). In addition to a gap in ownership, usage patterns also showcase inequity. Across all LMICs women are 20% less likely than men to use mobile internet; in India women are 50% less likely.^1^ These gender gaps in mobile phone use are more pronounced in rural areas and in regional pockets. In India, while household phone ownership in rural areas was only slightly lower than in urban areas (91% rural vs 93% urban) rural women’s access to mobile phones was far lower than their urban counterparts (42% rural vs 63% urban).^2^ In the central Indian state of Madhya Pradesh, where this study takes place, over 85% of rural households had phones, but fewer than 20% of rural women reported access.^2^ Additional components of identity intersect with the gender gap, exacerbating the marginalisation of women who are disadvantaged by economic, social and political injustice. In India, this means that the gender gap is higher for women in less educated, lower caste and poorer families.^3^

The gender gap in mobile phone use denies women equal access to the wide range of economic, social and health benefits of mobile phones. Mobile phone use can improve individual economic outcomes by promoting banking and increasing money transfers^4 5^ and by improving market performance.^6 7^ Mobile phones facilitate relationship maintenance,^8 9^ enable the growth of larger social networks, and increase social capital.^10^ Mobile phones can accelerate access to information and facilitate non-formal ongoing education^11^ and bring health benefits^2^ through facilitating access to outreach services, clinical care, appointment reminders, health information and follow-up services.^12^

Mobile phones are increasingly a requirement for modern civic participation. The Indian government is moving to an ‘integrated e-service delivery’ system across ministries, grounded in the digitalised unique citizen identification system (Aadhaar).^13^ Private banking and government financial entitlements are increasingly tied to mobile phones, which enable customers to receive notifications and access security codes. The Indian government’s open data commitment is being channelled through the MyGov portal, tying access to information and opportunities to participate in accountability efforts to digital access. The COVID-19 pandemic accelerated reliance on digital technology for education, health communication and other services.^14 15^

Women’s lower use of mobile phones is both a manifestation of past inequity and a driver of inequity going forward. Understanding this gender gap is fundamental to taking action to correct it. Yet common conceptualisations of the mobile phone gender gap stop at measuring access or use (eg, whether women use a phone or not, what they use the phone for) without contextualising use within a framework of determinants (eg, the factors that shape their phone use profile). India’s National Family Health Survey asked each female respondent if she ‘has a mobile phone that she can use’ but the concepts of ‘having’ and ‘being able to use’ were not defined or explored.^16^ While differential access and use between men and women is well established,^1^ this paper seeks to systematise and deepen our understanding of the drivers of these outcomes. To this end, our study illuminates the multifaceted determinants of the digital gender gap among married women and their husbands in rural Madhya Pradesh, India and presents a framework on the determinants of women’s phone use.

### Conceptual framework

**Figure 1:**
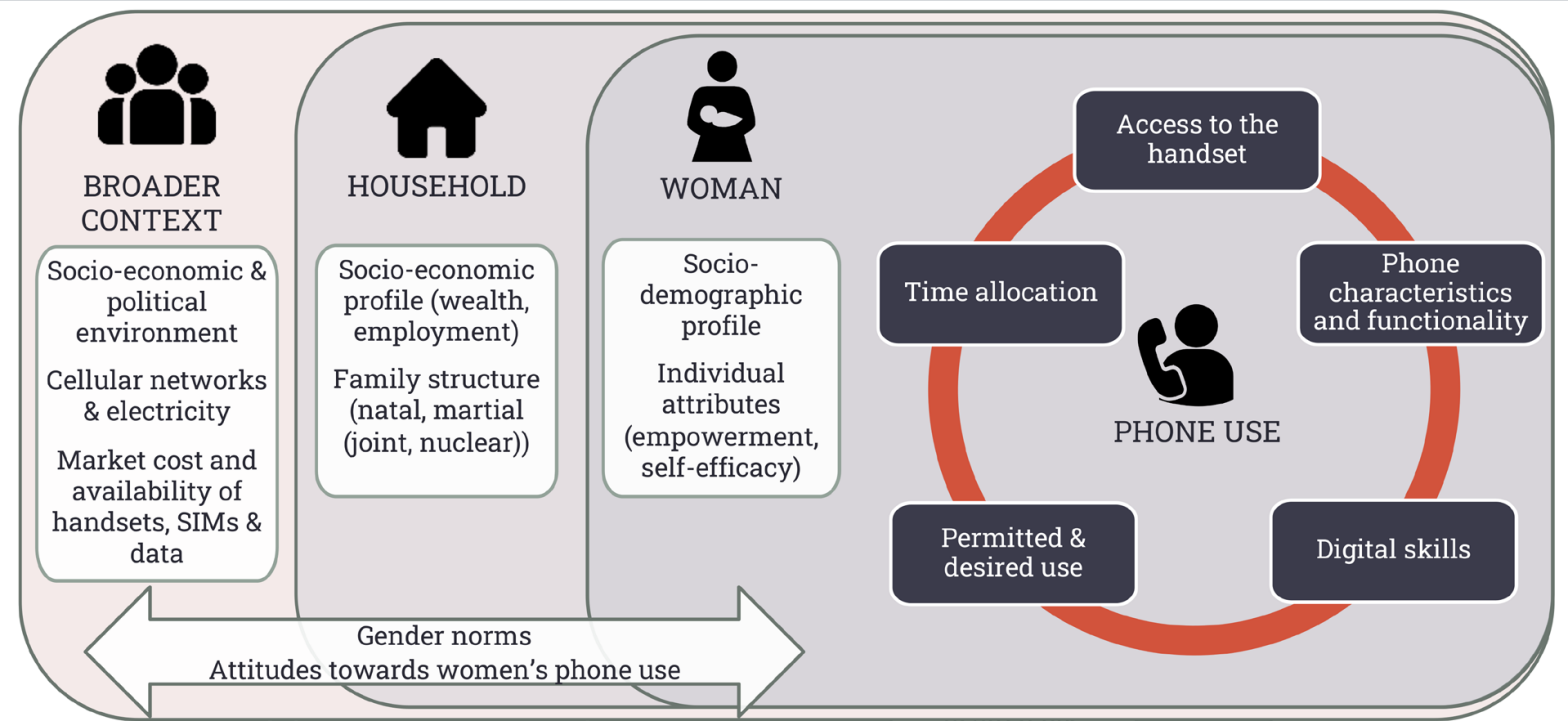
Determinants of women’s mobile phones use.

Our framework (figure 1) was developed iteratively from the theoretical literature on digital access and our research findings. It identifies five proximal determinants of women’s mobile phone use—access to the handset; phone characteristics and functionality; digital skills; permitted and desired use; and time allocation. These proximal determinants, which have been variously articulated in the literature as gender differences in access, extent of use, technical skills and social support in using technologies,^17 18^ are then positioned within the individual, household and broader context, underpinned by gender norms.

Gender norms and manifestations of these norms in atttudes about women’s phone use drive the digital divide.^19 20^ In Madhya Pradesh, patriarchal gender norms have been identified^21^ as: (1) domestic focus, wherein married women were expected to focus their energy on the maintenance of the home and family unit through cooking, cleaning and caring for children; (2) patriarchal exogamy, wherein on marriage, women join their husband’s family and leave behind their natal family; (3) purity, wherein married women must avoid any suspicion of sexual relations outside marriage and (4) subservience, wherein married women submit to the needs and wishes of their husbands and their in-laws. These norms drove collective attitudes that position phones as a risk to women’s reputation and as a potential distraction from caregiving. The extended Technology Acceptance Model (TAM2)^22^ and diffusion of innovations^23^ identify image (the effect of the technology on one’s social status) as a key driver of technology use. TAM2 also identifies job relevance and subjective norms (the individual’s thoughts on how important people in their life will view their behaviour) as cognitive and socially determined influences on technology use. Figure 1 adapts these influences to a gender-specific understanding of determinants of women’s phone use.

Broader contextual features that influence phone use include socioeconomic and political factors (including government policies or non-governmental interventions to improve phone accessibility),^19^ cellular networks, electricity and the market costs and availability of mobile phone handsets, subscriber identity module (SIM) cards and mobile internet data.^24–26^

Household-level norms and attitudes are shaped by the community and broader society as well as by the specific attributes of the woman herself. Each family will vary according to its internal dynamics and degree of adherence or resistance to these broader norms. Additional household-level determinants are the number of phones in the household and who owns them,^27^ the financial status of the family and employment status of family members, and the family structure.^20^ Additional phones in the household increase the possibility of female access. Family wealth affects the type of phones available in the home and the credit loaded on to the phone (talktime and mobile data). When phones are required for a family member’s employment, their access will be prioritised^20^; when employment separates family members (such as with migrant labourers), phone allocation will reflect the communication needs of the family. Finally, family structure will influence women’s phone access and use in terms of number of extended family members in the house who may support or hinder phone use, and children in the family who may seek phone access for entertainment or education.

The individual woman’s sociodemographic factors (particularly her age and education) and individual attributes (such as her empowerment and self-efficacy) serve as a third set of distal determinants of current phone use. A woman’s empowerment, that is, her ability to make strategic life choices,^28^ and self-efficacy, that is, her belief in her capability to organise and execute action,^29^ influences her mobile phone use through her power and ability to assert her needs and desires related to the phone. Education not only enables text-dependent and number-dependent phone uses, but interacts with women’s empowerment to increase women’s desire to own and use technology.

The elements of the framework can be reciprocal in nature, meaning that the distal and proximal determinants influence phone use, while phone use itself can influence these determinants. So, for example, gender norms that lead to a woman’s limited literacy, poor digital skills and low self-efficacy constrain how she uses the phone. But her ongoing use of a phone can expand her digital skills, increase her belief in her capabilities, and—although by no means linear nor guaranteed^30^—slowly, incrementally, shift gender norms.^31^

## Methods

### Study setting

Madhya Pradesh is a Hindi-speaking state in the centre of India with a population of approximately 84 million.^32^ Just 59% of women age 15–49 are literate, compared with 82% of men.^33^ Only 40% of women are employed outside the home, compared with 84% of men. Sixty-five per cent of women do not have any money that they can decide how to use. While 61% of currently married women participate in making decisions about their own healthcare, major household purchases and visits to their own family or relatives, 17% do not participate in making any of the three decisions. There are only 918 girls under the age of seven for every 1000 boys.^33^

### Data

This analysis of gender and digital access occurred within a larger impact evaluation of the Kilkari mHealth messaging programme in rural Madhya Pradesh, described and reported elsewhere.^34 35^ The data for this analysis were generated in the qualitative component of the Kilkari evaluation,^36^ which consisted of interviews with married women at 1-year post partum (n=29), who had been randomised to receive Kilkari during their pregnancy and whose call data records captured by the Kilkari IVR (Interactive Voice Response) system showed moderate to high Kilkari listenership. As often as possible, we also interviewed their husbands (n=23) and other family members (n=25), either one-on-one or in family group interviews (table 1 and online supplemental file 1, respondent profiles).

10.1136/bmjgh-2021-005596.supp1 Supplementary data

**Table 1:** Data collected, presented by research participants and by methodology

Research participants	N
Kilkari women	29
Husbands of Kilkari women	23
Mothers-in-law of Kilkari women	13
Other family members of Kilkari women	12
**Data collection methodology**	
One-on-one interviews	
In-depth interviews with Kilkari women	29
In-depth interviews with husbands of Kilkari women	10
Family group interviews (two to five family members of Kilkari women, including husbands in all but two cases)	15

Findings on the implications of women’s phone use on Kilkari exposure and impact are reported elsewhere.^36^ The Kilkari evaluation’s sample (n=5095 women) excluded pregnant women who did not have access to a phone,^34^ a group that consists of those from the poorest families with no household phone at all, and those in families with a phone but that did not allow the woman any access.^21^ Our qualitative sample was selected from within the larger Kilkari evaluation sample, thus, we similarly only spoke to women who had some access to phones (table 2). The possible implications of this sample are explored in the discussion section.

**Table 2:** Sample population, compared with Kilkari evaluation population and overall rural Madhya Pradesh

Characteristic	Qualitative sample (N=29)	Kilkari mHealth evaluation sample (n=5095)^34^	Rural madhya pradesh state
Household phone ownership	100% (n=29)	100%	86%^33^
Pregnant/postpartum women with access to a phone	100% (n=29)	100%	50%*
Literacy (could read a whole sentence)	48% (n=14)	56%	51%^33^
Poorest two wealth quintiles (Q1 and Q2)	52% (n=15)	40%	40%^33^
Highly marginalised caste (scheduled caste/schedule tribe)	45% (n=13)	17%	37%^33^

*In 2014–2015, only 19% of all women in rural Madhya Pradesh had access to a phone,^33^ however, among reproductive age rural women, phone access was estimated to be 40% in 2018^43^ and found to be 50% during the Kilkari household listing.^42^

In addition to topics related to the Kilkari mHealth programme, the interviews also covered family mobile phone use, gender dynamics around mobile phones within the home, cultural norms and community views on mobile phone use by men and women more generally, and how the stakeholders themselves engaged with mobile technology. Our findings on gender and phone use emerged from analysis of these domains within the interviews.

### Analysis

Analysis involved daily debriefs, coding and thematic analysis, and dyadic analysis of wife–husband pairs (see online supplemental file 2 for an example of the dyadic and thematic analysis). Analytic debriefs included detailed discussion of each respondent’s comments on a range of gender and digital use topics (such as mobile phone use, number of mobile phones in the home and their functionality, and social norms around the use of mobile phones). Coding and thematic analysis began with the development and application of a codebook. Text segments in the transcripts that were relevant to gender and phone use were tagged with the appropriate code. We then generated and read code outputs to identify themes on gendered determinants of phone use and access. To further map and contextualise our understandings of access to and use of phones, we re-examined transcripts as wife–husband dyads whenever possible (n=23 pairs) to compare determinants of mobile phone use between husbands and wives. Examining these themes and dyads in light of existing literature on gender and mobile phones enabled the iterative construction of our framework (figure 1, above).

10.1136/bmjgh-2021-005596.supp2 Supplementary data

### Patient and public involvement

The research participants were not involved in the design or reporting of this study, and the results have not been disseminated to them. However, the research was shaped by their priorities, experiences and preferences around gender and mobile phones through iterative probing and flexibility within our research domains.

### Findings

We first present overarching findings on how the women in our sample used phones and how this related to phone use among husbands. We then present the five proximal determinants of women’s unequal phone access and use from our framework. In the discussion we link these determinants to underlying patriarchal social norms.

#### Wide range in women and men’s mobile phone usage profiles but enduring gender gap

There was wide variation among respondents in terms of their self-reported engagement with mobile phones. Respondent phone access and use emerged through discussion as a complex phenomenon across multiple facets: what the respondent’s phone could do, what the respondent knew how to do, and what the respondent actually did on a regular basis. When examined in wife–husband pairs, the expected gender gap in phone emerged clearly (box 1).

Box 1 **Example of wife–husband phone usage profiles: complete reliance on husband**

**Figure 2:**
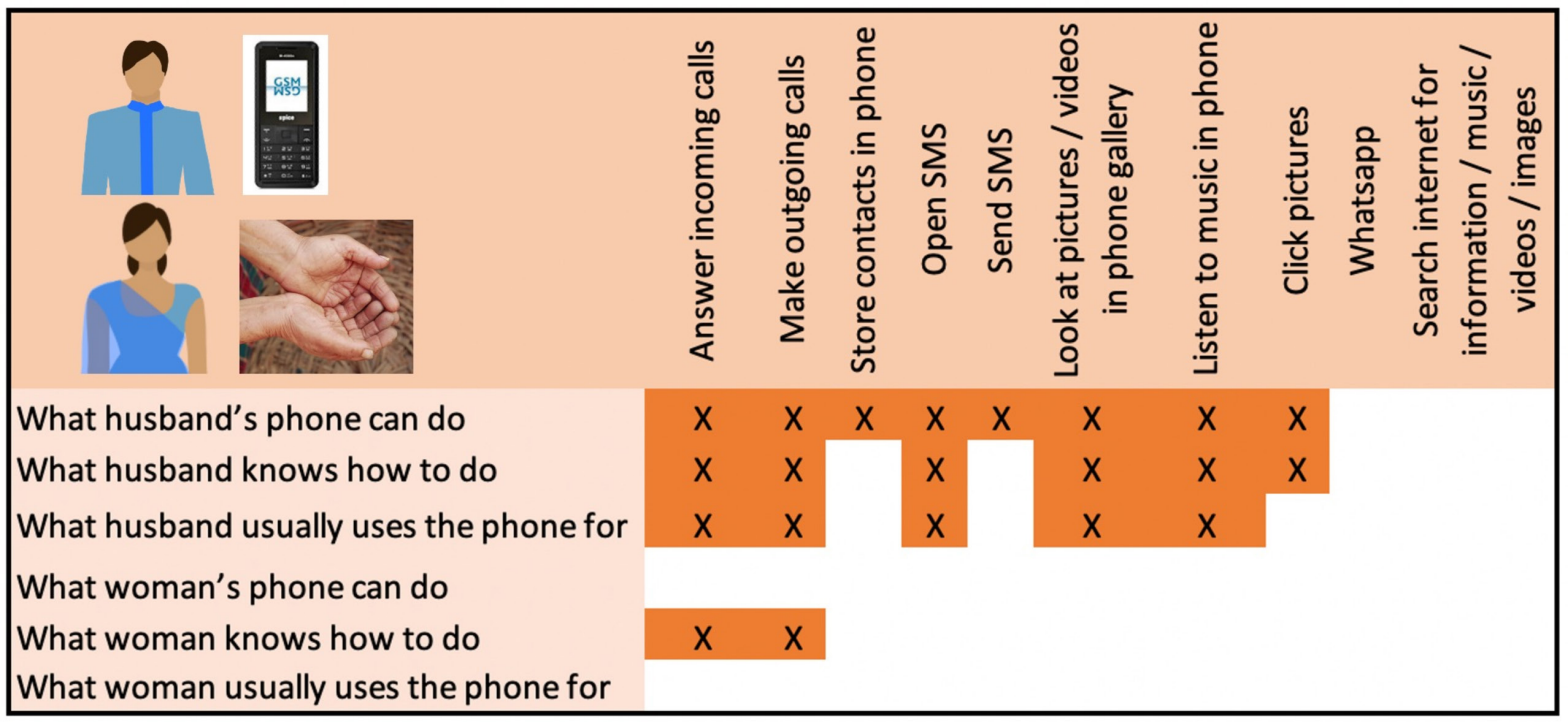
Dyadic comparison of phone use for WOM_26 and HUS_26.

WOM_26 and HUS_26 live in a nuclear family with their two young children. They are very poor (wealth quintile 1) and members of a marginalised scheduled caste group. HUS_26 owns a feature phone (button-type phone with a camera); WOM_26 does not have a phone (figure 2).

HUS_26’s phone is not internet enabled but he loads the memory card with music, images and videos and uses Bluetooth to share content with friends. He keeps it with him throughout the day when he leaves the house. WOM_26 has access to her husband’s phone only in the mornings and evenings when HUS_26 is home from work. She reports that she can answer incoming calls and make outgoing calls. However, since the phone is carried by her husband, he answers all incoming calls. If an incoming call is for WOM_26 and HUS_26 answers the call while home with her, he passes the phone to her. He also dials for her when she wants to make a call. She is not literate and is not able to store contacts or use SMS. She does not take photos or view any media on her husband’s phone.

Box 2 **Example of wife-husband phone usage profile: wife a basic user, husband super savvy**

**Figure 3:**
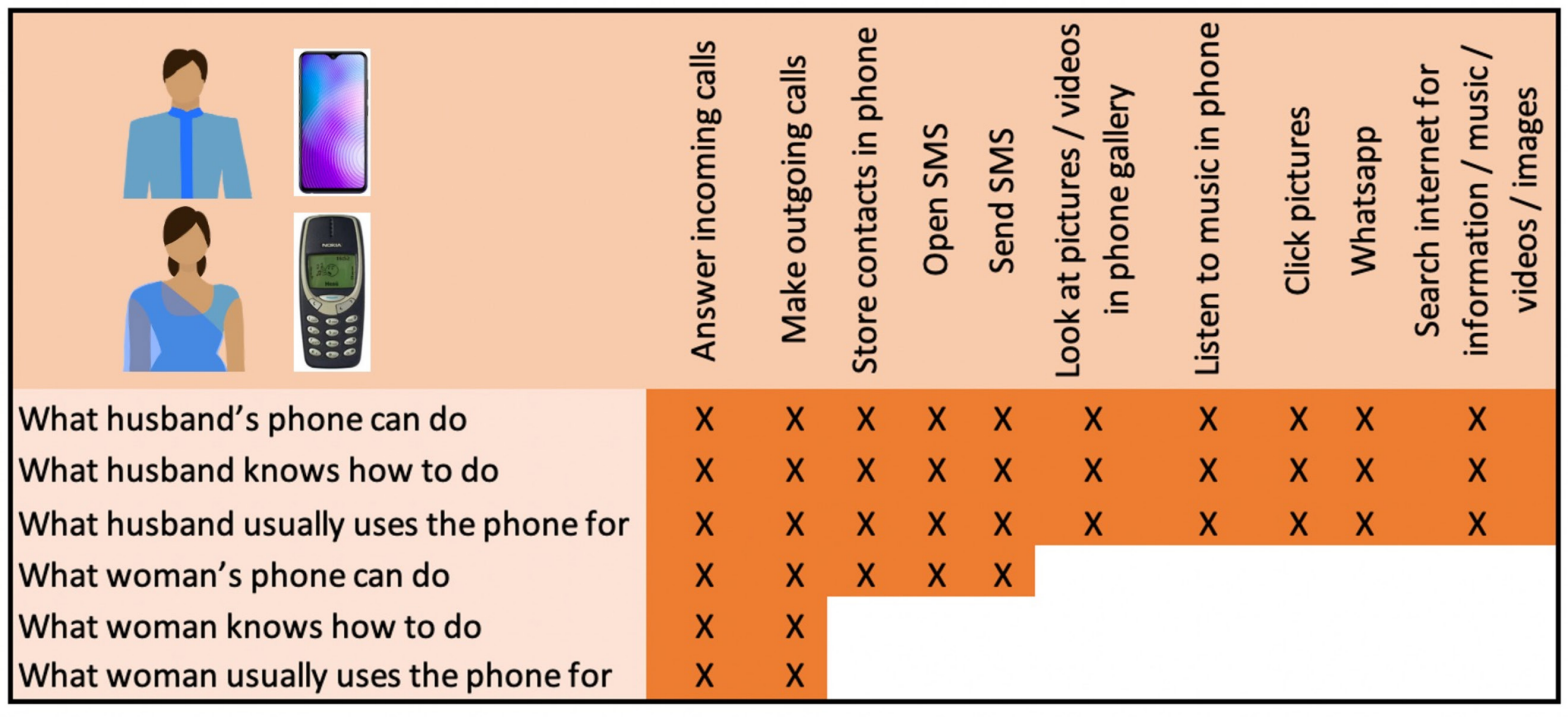
Dyadic comparison of phone use for WOM_12 and HUS_12.

WOM_12 and HUS_12 live with HUS_12’s parents and siblings and have one young child. They are wealthier (wealth quintile 4). HUS_12 owns a smartphone; WOM_12 owns a basic phone, which was given to her 4 years ago by her husband when he bought a new button phone with a camera for himself (figure 3).

HUS_12 recently bought himself a smartphone and uses many applications including Facebook and YouTube. WOM_12 is semiliterate and only uses the phone to pick up and dial calls, which she does regularly on her own.

Box 3 **Example of wife-husband phone usage profile: wife has high capability despite low literacy**

**Figure 4:**
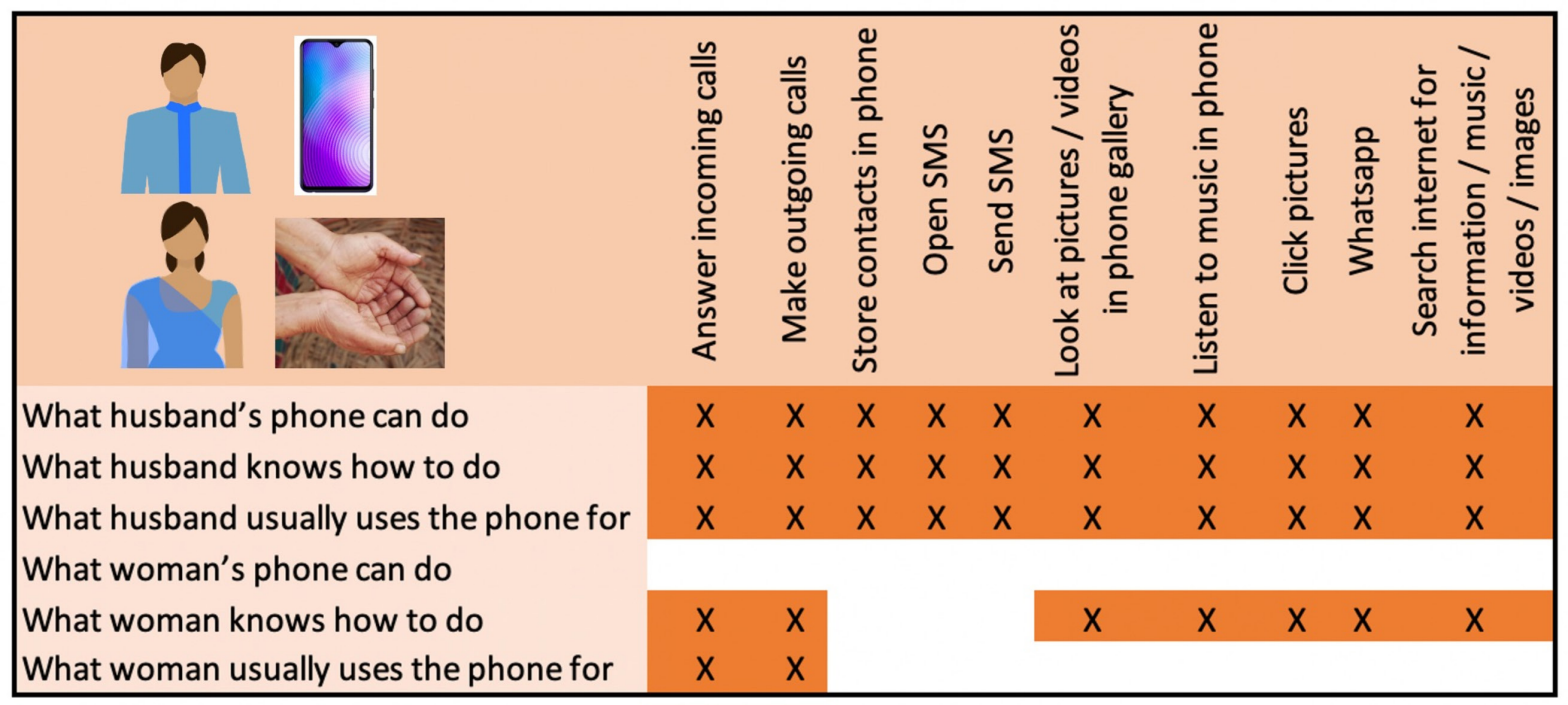
Dyadic comparison of phone use for WOM_09 and HUS_09.

WOM_09 and HUS_09 live with HUS_09’s parents and siblings and have one young child. They are wealthier (wealth quintile 4) and members of a scheduled caste group. HUS_09 owns a smartphone; WOM_09 does not have a phone (figure 4).

HUS_09 is a very savvy user, who has Facebook, Youtube, TrueCaller and other apps. WOM_09 knows how to use several features on her husband’s smartphone, although she only has access to his phone when HUS_09 is home from work and she only makes and receives calls when she accesses HUS_09’s phone. She is able to navigate the phone interface in many ways using visual clues (eg, to look at photos sent on WhatsApp) but she is not literate, which hinders her use of other features.

#### Women in our sample were ‘free’ to use phones

Respondents (both the women themselves and their family members) in our sample of moderate to high Kilkari listeners widely reported that married women’s use of mobile phones was not constrained by direct family control. Almost all our respondent families reported no overt monitoring or restriction on married women’s use of phones.

I: Do you need to take any permission for using the phone?

R: No, never.

I: How much freedom do you get for using the phone?

R: I have got full freedom. (WOM_03, technically owns the family’s brick phone but her husband keeps it with him throughout the day)

She doesn’t take any permission. She uses it according to herself. She sees things or listens to things. That’s it! (HUS_22, owns a feature phone that his wife borrows)

No one has any restriction like they cannot talk or anything like that. (HUS_28, owns a brick phone that his wife borrows)

Husbands also presented themselves as broadly supportive seeing their wives gain digital literacy. When asked by our researchers about whether their wives should receive mobile use training, including on e-commerce, husbands said they would support this training. There were two exceptions, wherein respondents explicitly stated that women were barred from handling the husband’s phone: in both cases (FAM_14 and FAM_17) the woman was not allowed to use her husband’s new smartphone beyond speaking on it after her husband dialled or picked up a call for her, although she had been allowed to handle the older phone. In HUS_17’s case, even after reporting that he did not allow his wife to handle his smartphone, he persisted in endorsing a liberal attitude towards women’s phone use by saying that his wife should use phone and that women should know how to use smartphone features.

#### Phone use limited by the ‘cage’ of proximal barriers

Despite most respondents reporting that women had no overt limitations on their phone use, they were nonetheless constrained by gender inequity across five areas, discussed in turn below: (1) permitted and desired use, (2) access to the handset, (3) phone characteristics and functionality, (4) digital skills and (5) time allocation.

##### Proximal barrier 1: permitted and desired phone use

Women’s use of phones was limited by narrow family expectations and limited personal desire in terms of what the phone could be used for. Phones were readily provided to women, and family members did not monitor who women were speaking to, because it was well accepted that women only use them to call their husbands and natal family members (parents and siblings).

Who else would I talk to? [Just] Ma and Papa. (WOM_05, owns a brick phone)

R : Yes, we have to ask for his [father-in-law’s] phone. He gives it then. He doesn’t ask that where we want to speak.

I : He doesn’t ask you?

R : He knows that I will speak to his son. (WOM_27, borrows her husband’s smartphone when he is home; uses her father-in-law’s brick phone when her husband is not home)

With a few exceptions, women were not expected to and did not express any desire to use the phone for communication with friends or for employment, two common phone uses reported by men. Most women reported never using the phone to speak to anyone beyond family, explaining that they did not have friends and were not employed outside the home.

I: Similarly, do you have any friends from school you speak to sometimes?

R: No, I don’t even know what a friend is like. (WOM_18, owns her own brick phone)

I: Is it that you don’t have a friend or you don’t call them?

R: I don’t have a friend. Also I do not talk to anyone. (WOM_28, borrows her husband’s brick phone)

There were exceptions: WOM_10 (borrows her husband’s feature phone) reported sometimes communicating with friends by phone and WOM_03 (technically owns her own phone but her husband keeps it with him) reported using the phone to coordinate her work as a cook in a school. WOM_10’s engagement with friends may have been driven by the fact that she was pursuing a bachelor’s degree, and was one of only two women in our sample who had more than 12 years of schooling. WOM_03 was our only female respondent with salaried work outside the home. While most female respondents said that they did not speak to health workers by phone, two women (WOM_05, WOM_18, both own their own brick phones) said that they did.

In terms of entertainment, about half the women reported not consuming any music, videos, photos or social media on the phone. Among those who did access entertainment on the phone, many framed this use as low importance, infrequent and often for the benefit of their children. Men on the other hand, spoke of entertainment on the phone as a central reason for phone use.

R: He [HUS_14] listens to music. He watches movies as well if there is time. And if not, he goes to work, he is at work the whole day.

I: And what about WhatsApp, Facebook, and Tik Tok?

R: Yes, he uses them.

I: He uses them?

R: Yes.

I: And what about you? Do you listen to music or watch videos?

R: No.

I: You do not watch movies? He watches them alone?

R: No. [Laughs] I am busy with my son. I only care for him. (WOM_14, borrows her husband’s smartphone)

I: What kind of movies do you like?

R: Just like that I get them in my phone.

I: Hindi movies, Bhojpuri movies. What kind of movies you like?

R: I like all them. I watch bits of all movies.

I: Anyone else also watches?

R: No.

I: Like there are three people in your family. Like your wife?

R: There is television at home. So she watches that. (HUS_15, owns a feature phone that his wife borrows)

##### Proximal barrier 2: access to the handset

Women’s use of phones was limited by their dependence on men to buy or lend them phones and by their frequent physical distance from phones. While women who owned their own phones had much greater access to them than women who borrowed their husband’s, women lacked the financial autonomy to buy themselves phones. They received phones as hand-me-downs or gifts from male family members (usually their husbands).

I : Who bought this phone?

R : My husband.

I : You husband.

R : Who else will buy the phone? [laughs] (WOM_05, owns her own brick phone)

Women were not seen to need phones as much as men because women did not use phones to access employment (with the exception of WOM_03, the school cook). Thus women’s phone ownership was seen as a luxury, and if her husband’s phone broke he would take over her phone.

Women also spent more time physically away from phones, which limited their capacity to answer incoming calls. While men kept their phones in their pockets, particularly when outside the home, women who owned their own phones tended to keep their phones on surfaces and left them in one place when moving around the home and compound. When women shared phones with their husbands, the phone was generally kept by the husband throughout the day and she had to ask him to access it. Respondents emphasised that she was not asking permission but simply asking for the phone to be handed to her—but this was nonetheless a barrier.

I: Ok. And who has the mobile for the maximum time?

R: My husband has it.

I: Your husband, ok. The mobile which your husband has, when can you use it?

R: I use it only if I get time. I use it to call my mother, otherwise I don't use it. (WOM_09, borrows her husband’s smartphone)

No, I don’t keep it [the mobile] with me all the time. I leave it anywhere in the house. (WOM_07, owns her own brick phone)

##### Proximal barrier 3: phone characteristics and functionality

Among couples where both spouses had phones, women’s use of digital technology was limited by the fact that their personal phones could perform fewer features than their husbands’. Some women reported knowing how to use advanced features, such as how to navigate WhatsApp or view movies, but rarely or never executing these skills because they were constrained by the absence of these features on their phones. For example, WOM_13 knew how to play songs and take pictures when she had access to her husband’s phone but could not execute these functions on her phone, since it did not have a memory card. None of the women in our sample who owned their own phones had a better phone than her husband and very few had phones with equal features. For example, WOM_06, WOM_07, WOM_12, WOM_19, and WOM_23 all had simple brick phones while their husbands had internet enabled smart or feature phones. Even among our respondents where both the husband and wife had simple button phones (FAM_05, FAM_11, FAM_13, FAM_18, and FAM_25), several husbands (HUS_05, HUS_11, HUS_13) had memory cards added to their phones while their wives did not.

Women who owned their own phones had frequent connectivity gaps due to ‘zero balance’ (no money loaded on the phone) on their phones and almost all respondents reported that less financial credit was loaded onto women’s phones. Women did not ‘top up’ the financial credit on their own phones because they had low financial autonomy and were encouraged to stay in the home.

I: So, do you recharge her phone, or does she do it on her own?

R: No, I do it. I am the earner, she is a lady, a housewife. [Laughs] (HUS_24, owns his own smartphone that his wife borrows)

Since men were responsible for topping up credit women could only ask and remind men to add credit to their phones. They reported that men often did not top up their phones in a timely manner, leaving them without functioning phones for periods of time ranging from a few days to weeks.

##### Proximal barrier 4: digital skill

Women’s phone use was constrained by more limited digital skill, for example, lower ability to navigate and use the full range of available features on the mobile phone. Both male and female respondents presented digital capacities across the spectrum from highly savvy (able to use social media and information platforms, as well as saving contacts, texting, taking photos and using the calculator, BlueTooth and hotspots) to basic (just making and receiving calls). However, women more often spoke of not knowing how to access features on the phone and female respondents often said that they relied on their spouses to perform some functions for them. This lower digital literacy was closely tied to women’s lower rates of literacy.

However, lower digital literacy was driven not only by lower literacy but also by lower confidence, discouragement from family, and fewer opportunities to gain digital skills. For example, WOM_14 knew how to dial and receive calls on the brick phone she used to borrow from her husband. However, he recently upgraded to a smartphone and she reported that she is unable to perform any tasks on this new phone. Her husband dials for her now and has not given her any opportunities to learn how to navigate the new phone. Moreover, she reported that she does not want to learn to use the new phone, because if she learns to use it she will become interested in it, and this would be a problem because she does not have access to it.

R: He does not give it.

I: Okay, do you wish to learn?

R: No. [Smiles]

I: No?

R: No, he does not give it to me and I do not even feel like learning to use it.

I: Okay.

R: If I do, I will get interested in it. Now, since I do not have it so I do not learn to use it.

I: Okay, that is the reason? That you do not have it?

R: Yes. And we do not have much money that we buy it. (WOM_14, borrows her husband’s smartphone)

WOM_26 explained that her lack of education prevented her from using the phone, despite the fact that she was able to read and that some features, like the camera or watching a video, are somewhat accessible even without literacy and were used by her husband, who was himself only semiliterate.

I: Ok. What all do you know in the phone? Can you use it?

R: I haven’t studied sister so how can I.

I: Ok. So, if you have to dial on the phone then your husband does it?

R: Yes.

I: […] Do you listen to songs?

R: I never listen to songs.

I: Have you ever seen a video or clicked a picture?

R: Nothing. (WOM_26, borrows her husband’s feature phone)

##### Proximal barrier 5: time allocation

Women’s use of phones was limited by scarcity of leisure time as well as norms that hindered the use of phones for leisure. It was widely accepted that men used the phones for ‘time pass’ (entertainment).

I : Do you use WhatsApp?

R : Yes.

I : Facebook?

R : I do.

I : Ok, you use Facebook. That means you use all these?

R : Yes.

I : Do you ever watch videos on YouTube?

R : Yes, I watch on YouTube. Like, if I want to watch any movie. I want to pass time. And songs. All these things. (HUS_19, owns his own smartphone and his wife has her own brick phone)

Respondents emphasised how busy women were, and explained that they have limited time available to use phones.

R: [I use the phone when] he returns in the evening.

I: You do not get time in the morning?

R: In the morning, I make breakfast, there is work. (WOM_14, borrows her husband’s smartphone)

I use it only if I get time. I use it to call my mother, otherwise I don't use it. (WOM_09, borrows her husband’s smartphone)

Women’s domestic work in rural Madhya Pradesh was labour intensive. However, gender inequity on time allocation extended beyond a lack of leisure time: even when women had time to rest, they could not be seen to be spending this time on the phone, unless they were speaking to their family members. Respondents explained that women could be negatively judged for spending ‘too much’ time on the phone because they may be seen to be shirking domestic responsibilities or accused of inappropriate behaviour, including gossip and infidelity.

There is my Jeth’s [husband’s elder brother’s] daughter-in-law. I don’t know much about her. […] I have mostly heard that she keeps sitting with the phone throughout the day and doesn’t do household chores. […] They say that bahu [daughter-in-law] keeps using the phone and I have to do all the work. [laughs] (WOM_03, technically owns the family’s brick phone but her husband keeps it with him throughout the day)

R: She doesn’t talk [to friends or health workers].

I: Ok. Why doesn’t she talk to anyone? What is the reason for not talking?

R : I don’t know. She doesn’t talk too much. Zada faltu kisi se baat nahi karti. [She doesn’t talk useless things much to anybody]. She is busy with her work like taking care of the children, cleaning the house. She does all this. (HUS_16, owns his own brick phone)

I: Ok. What if a woman has her own phone? What do people think of her in the neighbourhood?

R: They think that she uses it for spurious purposes […] [That]

she engages with bad persons. She indulges in obscene talks and some people take advantage of that. (HUS_29, owns his own smartphone but it is currently broken and he is using a brick phone; his wife currently has no phone because her smartphone broke)

## Discussion

This paper presents a framework of the multifaceted determinants of the gender gap in mobile phone use derived iteratively from existing literature and qualitative research with mothers of young children in rural central India and their families. Despite assurances from women and their husbands that women were ‘free’ to use the phone as they wished, their actual engagement with phones was tightly constrained due to barriers across five proximal determinants: First, families expected married women to use phones solely to communicate with their natal families and husbands, and women themselves expressed a desire to use the phone only for this purpose. Second, women had limited physical proximity to the phone and depended on men to buy them phones, resulting in lower rates phone ownership, echoing other research in India.^27^ Third, women frequently had older phones with fewer features than their husbands, in line with previous studies,^1^ had less financial credit loaded on the phone, and experienced frequent ‘zero balance’ periods because they had to rely on men to load credit on their phones. Fourth, women generally had lower digital skill, which was driven by lower literacy and numeracy, as well as lower confidence and fewer opportunities to learn to use newer phone technology. Fifth, women allocated limited time for engaging with the phone, which was explained as a product of their heavy domestic burdens and, echoing others,^21 37^ social norms that made it inappropriate to be seen spending leisure time on the phone.

Patriarchal gender norms of domesticity, subservience, purity and family relationship maintencance^21^ underpin these determinants. Domesticity and subservience norms in particular drove women’s low financial autonomy and inequal access to employment outside the home.^20 27^ These manifestations in turn shaped a reality where families understood uninterrupted male access to a functional phone as essential, since men’s access to employment was often linked to the phone, while women’s phone use could occur sporadically on lower quality devices, since it did not generate income for the family and in fact could distract women from domestic responsibilities. Purity norms have been found to be particularly salient for women leading up to marriage^21^ but even our married respondents noted the importance of ensuring that phone use could not be misconstrued as improper in any way. The ways in which mobile phones serve as sites of power or control, and the risks and benefits associated with women’s phone use that transgresses gender boundaries have been discussed elsewhere,^38–40^ and merit further exploration.

The framework presented here highlights the interconnected nature of barriers to women’s mobile phone use and points towards the need for interventions across multiple areas. For example, interventions that give women mobile phones can increase ownership and proximity, although men in the family may still appropriate these phones. When in possession of the phone, women may still lack the skills to use many of the phone’s features, may lack the physical and financial autonomy to go to the shop and add financial credit to the phone, and will continue to navigate family and community expectations and their own personal desire that constrains the bounds of appropriate phone use. Interventions addressing multiple proximal barriers show promise in boosting and diversifying women’s phone use towards broader economic and social development: a female-focused microloan programme that relied on mobile banking expanded women’s desired use for their phones and intermediary loan officers encouraged women and taught them the requisite digital skills.^41^ And more broadly, intervening at the level of proximate barriers to boost phone use will have a deeper impact on women’s empowerment when patriarchal gender norms are challenged in diverse ways across many domains.^30^ The framework also points towards better measurement in the digital space, wherein surveys could assess each of the proximal determinants to illuminate constraints on women’s actual use of mobile phones.

This study reports on the determinants of mobile phone access and use for a specific type of woman in rural central India: married women who have a 1-year-old child, who self-reported that they have access to a mobile phone, and whose mobile number enrolled in Kilkari showed moderate to high listenership (indicating that someone in the household was picking up incoming Kilkari calls). Women who reported that they had no access to mobile phones were excluded from this study: this excludes women in households without a phone, which are the poorest families,^33^ and households with a phone wherein pregnant women lacked access (approximately 50% of rural households in Madhya Pradesh in 2018).^42^ These latter women are likely in the more conservative households; women in households that own phones who lack personal access have been found to be significantly less empowered across a range of measures.^21^

Taken as a whole, our sample portrays a more empowered or progressive version of women’s phone use compared with the norm in the region, which is striking considering that even among this sample we nonetheless identified extensive barriers to women’s phone use. Further research is needed to assess the resonance of the determinants of mobile phone use identified in this paper in relation to the lived realities of women in households with a phone who have no access at all, adolescent girls, urban women and older women (eg, those with grown children). While our sample included just one women employed outside the home, additional research among employed women would enable deeper understanding of the linkage between women’s paid work and phone use. In addition to assessing this framework among other populations of women, there is a pressing need to apply these determinants to exploring possibilities for change.

## Conclusion

As both a manifestation and potential exacerbator of gender inequity, policy and programmes must take multipronged approaches to limit the damage of the gender gap and ultimately eliminate it. Thus, any initiative seeking to harness the potential of mobile phones, including in the health, education, governance and financial sectors, must simultaneously bolster non-phone reliant systems as well, to avoid leaving behind already-marginalised women. Furthermore, phone-based initiatives should include programming that maximises accessibility to women experiencing many of the barriers described here. Assumptions of widespread social media and smartphone use, high digital skill, literacy and numeracy, and unfettered access and functionality should be interrogated. Simple audio-based programming (of which Kilkari is an example) should be emphasised. Campaigns to increase the acceptability of expanded forms of phone use among women may prove essential not only to increase community and household buy-in, but also to broaden women’s own vision of the phone’s possibilities.

## Data Availability

Data are available on request. Data for this study consist of qualitative interview transcripts. Uploading all transcripts for open availability would compromise our ability to fully mask participant details. However, we are happy to share anonymised portions of these transcripts on reasonable request.
