# Supplementary material for: Freedom within a cage: how patriarchal gender norms limit women’s use of mobile phones in rural central India
Source: BMJ Glob Health. 2021 Sep 22;6(Suppl 5):e005596. doi: 10.1136/bmjgh-2021-005596 (PMC8461288; doi:10.1136/bmjgh-2021-005596)
Supplement: Supplementary data [file bmjgh-2021-005596supp001.pdf]

Supplementary file 1. Respondent profiles

Respondent	Phone	Wealth	Literacy*	Caste	Fam Struct	Age
WOM_01	Does not own, husband has a smartphone which is slightly broken	Poor (Q2)	Literate	OBC	Extended	23
WOM_02	Owns a brick phone; husband's phone type not clear	Wealthier (Q5)	Literate	Gen	Extended	31
WOM_03	Technically owns a brick phone but the husband has appropriated it	Poor (Q2)	Literate	Gen	Nuclear	38
HUS_03	Brick phone (see above)	Poor (Q2)	12th	Gen	Nuclear	43
WOM_04	Does not own	Poor (Q2)	Literate	Gen	Extended	32
HUS_04	Owns a brick phone which is out of order	Poor (Q2)	Master's	Gen	Extended	42
WOM_05	Owns a brick phone	Poor (Q1)	Illiterate	SC	Nuclear	23
HUS_05	Owns a brick phone with a memory card	Poor (Q1)	8th	SC	Nuclear	29
WOM_06	Owns a brick phone with a memory card	Poor (Q2)	Literate	OBC	Extended	25
HUS_06	Owns a smartphone	Poor (Q2)	8th	OBC	Extended	32
WOM_07	Owns a brick phone	Wealthier (Q5)	Literate	Gen	Extended	22
HUS_07	Owns a smartphone	Wealthier (Q5)	12th	Gen	Extended	22
WOM_08	Does not own	Poor (Q2)	Illiterate	OBC	Extended	23
HUS_08	Owns a brick phone which is out of order	Poor (Q2)	8th	OBC	Extended	28
WOM_09	Does not own	Wealthier (Q4)	Illiterate	SC	Extended	21
HUS_09	Owns a smartphone	Wealthier (Q4)	B.A.	SC	Extended	26
WOM_10	Does not own	Middle (Q3)	Literate	OBC	Extended	21
HUS_10	Owns a feature phone	Middle (Q3)	9th	OBC	Extended	24
WOM_11	Owns a brick phone	Wealthier (Q4)	Semi-literate	Gen	Extended	24
HUS_11	Owns a brick phone with a memory card	Wealthier (Q4)	9th	Gen	Extended	26
WOM_12	Owns a brick phone	Wealthier (Q4)	Semi-literate	OBC	Extended	21
HUS_12	Owns a smartphone	Wealthier (Q4)	11th	OBC	Extended	22
WOM_13	Owns a brick phone	Poor (Q2)	Illiterate	OBC	Nuclear	27
HUS_13	Owns a brick phone with a memory card	Poor (Q2)	9th	OBC	Nuclear	32
WOM_14	Does not own, husband has a smartphone	Wealthier (Q4)	Illiterate	OBC	Extended	24
WOM_15	Does not own	Poor (Q2)	Illiterate	SC	Nuclear	23
HUS_15	Owns a Jio feature phone	Poor (Q2)	4th	SC	Nuclear	27
WOM_16	Does not own	Poor (Q2)	Illiterate	SC	Extended	28
HUS_16	Owns a brick phone with memory card	Poor (Q2)	5th	SC	Extended	30
WOM_17	Does not own	Poor (Q2)	Semi-literate	ST	Extended	20

Respondent	Phone	Wealth	Literacy*	Caste	Fam Struct	Age
HUS_17	Owns a smartphone	Poor (Q2)	8th	ST	Extended	24
WOM_18	Owns a brick phone with memory card	Wealthier (Q4)	Semi-literate	SC	Nuclear	25
HUS_18	Owns a brick phone with memory card	Wealthier (Q4)	3rd	SC	Nuclear	40
WOM_19	Owns a brick phone	Middle (Q3)	Literate	ST	Extended	23
HUS_19	Owns a smartphone	Middle (Q3)	12th	ST	Extended	30
WOM_20	Owns a brick phone, husband's phone unclear (interview cut short)	Middle (Q3)	Literate	ST	Extended	22
WOM_21	Owns a brick phone with a memory card	Poor (Q1)	Illiterate	ST	Extended	24
FAM_21	MIL & FIL do not own, Chachi (Owns) – husband not available and phone status unclear	Poor (Q1)	MIL & FIL illiterate, Chachi 5th	ST	Extended	**
WOM_22	Does not own	Middle (Q3)	Literate	OBC	Extended	26
HUS_22	Owns a Jio feature phone	Middle (Q3)	10th	OBC	Extended	27
WOM_23	Owns a brick phone	Poor (Q2)	Literate	SC	Extended	21
HUS_23	Owns a smartphone	Poor (Q2)	6th	SC	Extended	22
WOM_24	Does not own	Middle (Q3)	Literate	SC	Extended	32
HUS_24	Owns a smartphone	Middle (Q3)	Master's	SC	Extended	32
WOM_25	Owns a brick phone	Middle (Q3)	Literate	OBC	Extended	19
HUS_25	Owns a brick phone	Middle (Q3)	8th	OBC	Extended	27
WOM_26	Does not own	Poor (Q1)	Illiterate#	SC	Nuclear	25
HUS_26	Owns a feature phone	Poor (Q1)	8th^	SC	Nuclear	30
WOM_27	Does not own	Wealthier (Q4)	Illiterate	General	Extended	24
HUS_27	Owns a smartphone	Wealthier (Q4)	No data	General	Extended	Don't know
WOM_28	Does not own	Poor (Q2)	Illiterate	SC	Extended	26
HUS_28	Owns a brick phone	Poor (Q2)	No data	SC	Extended	No data
WOM_29	Had a smartphone but it broke	Poor (Q2)	Literate	OBC	Extended	22
HUS_29	Owns a smartphone but it broke so is currently using a brick phone	Poor (Q2)	8th	OBC	Extended	26

* Literacy was assessed for all the women during the quantitative baseline Kilkari survey. A woman was considered literate if she was able to read the whole sentence presented to her on a card during the quantitative survey. She was considered semi-literate if she could read only parts of the sentence. She was considered illiterate if she could not read the sentence at all. Husband literacy was not assessed. We asked them about their schooling during the qualitative interview and present their schooling attainment.
** respondent did not know
# the baseline quantitative survey reported that she was literate but in the qualitative interview she told us that she was not literate

^ although he has 8 years of education, during the qualitative interview he referred to himself as only semi-literate
