# Supplementary material for: Freedom within a cage: how patriarchal gender norms limit women’s use of mobile phones in rural central India
Source: BMJ Glob Health. 2021 Sep 22;6(Suppl 5):e005596. doi: 10.1136/bmjgh-2021-005596 (PMC8461288; doi:10.1136/bmjgh-2021-005596)
Supplement: Supplementary data [file bmjgh-2021-005596supp002.pdf]

Supplementary file 2: Example of dyadic and thematic analysis for the theme: Proximate barrier – phone characteristics and functionality

<table>
<tr><th>Dyads and quotations related to phone characteristics and functionality</th><th>Sub theme</th></tr>
<tr><td>
Among dyads where both spouses owned a phone in all but two cases, the husband had a phone with more advanced characteristics and higher functionality.
<table>
<tr><td colspan="2">Unequal pairs: husband had better phones</td></tr>
<tr><td>DYAD_05</td><td>Woman owns a brick phone, husband owns a brick phone with a memory card</td></tr>
<tr><td>DYAD_06</td><td>Woman owns a brick phone with a memory card, husband owns a smartphone</td></tr>
<tr><td>DYAD_07</td><td>Woman owns a brick phone, husband owns a smartphone</td></tr>
<tr><td>DYAD_11</td><td>Woman owns a brick phone, husband owns a brick phone with a memory card</td></tr>
<tr><td>DYAD_12</td><td>Woman owns a brick phone, husband owns a smartphone</td></tr>
<tr><td>DYAD_13</td><td>Woman owns a brick phone, husband owns a brick phone with a memory card</td></tr>
<tr><td>DYAD_19</td><td>Woman owns a brick phone, husband owns a smartphone</td></tr>
<tr><td>DYAD_23</td><td>Woman owns a brick phone, husband owns a smartphone</td></tr>
<tr><td colspan="2">Exceptions: equal pairs</td></tr>
<tr><td>DYAD_18</td><td>Owns a brick phone with memory card, husband also owns a brick phone with memory card</td></tr>
<tr><td>DYAD_25</td><td>Owns a brick phone, husband also owns a brick phone</td></tr>
<tr><td colspan="2">There were no dyads where women had better phones than their husbands.</td></tr>
</table>
** In the remaining dyads, not listed in the chart above, the woman has no phone and the husband has a phone. So her unequal use of the phone was not limited by the device itself but by access to her husband's phone, as discussed in proximate barrier – phone access.
</td><td>Women's phones could perform fewer functions than their husbands' because of

handset limitations</td></tr>
<tr><td>
Excerpt – Document: KL_WOM_06_20190913_AS.docx,
I: Ok. You don't operate WhatsApp and like you said you don't recharge your phone [telecom credits] only a balance of 35 is maintained.
R: **Yes, I only get a recharge of Rs 35. Otherwise what do I need it for.**

Excerpt - Document: KL_FAM_18_20190910_AJ.docx, Position: 2769-3179
I: For how much you recharge?
R: Suppose for Rs. 30/- it is free for 28 days.
I: Ok, in this you recharge for **Rs. 30**/- and it is 28 days free?
R: Hmm…
I: And for her?
R: **It is for her only. For me it is 199/- for 28 days**
I: And you are telling that the amount is less for her?
R: Yes.

Excerpt - Document: KL_FAM_24_20190912_AS.docx, Position: 3533-4770
I: Okay. How much do you recharge on it?
R: Recharge…whatever is free for the month, 129 or 169.
I: **And is there internet in the 129 recharge?**
R: Yes, we get 2GB in 1 month.
(05:05) I: Okay. And is there internet on didi's phone?
R: No, on my phone.
[…].
I: Okay. And when you recharge her phone, how much amount of balance is done per month?
R: **35**.
I: 35? Okay. And does it last for the whole month?
R: Because it is free on my phone, **if there is any need, she can call from my phone**, it is there because of some emergency. And other than an emergency, if we do not recharge for 35, both incoming and outgoing will become invalid.

Excerpt - Document: KL_WOM_01_20190827_AJ.docx,
I: Do you use internet on it?
R: **I do not use it regularly. If there is some need, then I recharge it for internet.**
</td><td>lower financial recharges on women's phones</td></tr>
</table>

Dyads and quotations related to phone characteristics and functionality	Sub theme
Excerpt - Document: KL_WOM_01_20190827_AJ.docx,<br>I: Does it happen that the phone is without any balance?<br>R: Yes, it happens.<br>I: Outgoing or incoming services have been barred.<br>R: Yes, it happens […]**But after 10 or 5 days, the phone is recharged.**<br><br>Excerpt - Document: KL_WOM_05_20190828_NP.docx,<br>I : Does it ever happen sister that since there was no money in the phone, so you were not able to speak. I am talking about your phone.<br>R : **When money is not there in my phone then how can I talk. It will be cancelled and we won't be able to talk.**<br><br>Excerpt - Document: KL_WOM_06_20190913_AS.docx, Position: 3449-3825<br>I: How much balance remains in your phone?<br>R: There is only Rs 35 balance. When I go and get a recharge done the balance doesn't remain only. Everyone calls me, my brother, it's all free.<br>I: Ok. So, they only call you.<br>R: They call me. **Calling is free on my husband's phone so I call from his mobile. There is no work on my mobile.**<br><br>Excerpt - Document: KL_WOM_09_20190901_AS.docx, Position: 4925-5882<br>I: Does it ever happen that the balance in the phone is zero and you can't call anyone? Does that ever happen? Or does your phone always have balance?<br>R: Yes, it happens many times, when we don't have money. We are poor people. When we don't have money, we don't recharge it and when we get money, we recharge it.<br>I: Ok. So if the phone has zero balance, can you still receive a call on the phone?<br>R: **If there is no balance, it comes for 2 to 3 days and after that it stops coming.**<br><br>Excerpt - Document: KL_WOM_13_20190904_AS.docx, Position: 2865-3387<br>R: Didi, there is no balance. **There was no balance and for a month I didn't recharge**. **I used to talk using Bhaiyya's phone to call mummy or anywhere else**. Using Bhaiyya's phone. This phone was not recharged.<br><br>Excerpt - Document: KL_WOM_15_20190905_AJ.docx, Position: 7329-7730<br>R: Jab paisa khatam ho jaata ha, tab nahin lagta hai(When it runs out of money, then I am not able to call from it).<br>I: You are not able to make a call.<br>R: yes.<br>I: **How many times does it run out of balance?**<br>**R: Monthly.**<br><br>Excerpt - Document: KL_FAM_18_20190910_AJ.docx, Position: 8942-9490<br>I: Hmm….anytime it has happened that your phone has been shut down due to lack of money, it has not been recharged. Anytime it has happened that your balance has been over?<br>R: Hmm…**her phone gets shut down sometimes but my phone never.**<br><br>Excerpt - Document: KL_WOM_23_20190912_MS.docx, Position: 6514-8312<br>R: Until we can recharge it again, it will stay at zero balance.<br>I: Okay so did it happen sometimes?<br>R: Yes, and the sim gets blocked. […]<br>I: So, for how many days does the phone stay blocked with no balance?<br>[00:10:02.07] R: **The first time, it was blocked for 10 to 15 days**. This time, it was 2 or 3 days.<br><br>Excerpt - Document: KL_WOM_25_20190913_MS.docx, Position: 3248-3828<br>R: **there is no balance then the sim stops working**. […] when there is money then do the recharge. When not then do not do…	Zero balance periods common for women
Excerpt - Document: KL_WOM_02_20190827_NP.docx,<br>I: And who recharges your phone?<br>R: **My husband does it.**<br>Excerpt - Document: KL_WOM_28_20190914_SY.docx, Position: 5293-6400<br>I: Okay. Who recharges the phone balance?<br>R: **My husband.**	Women rely on men to top up the phone

Dyads and quotations related to phone characteristics and functionality	Sub theme
Excerpt - Document: KL_WOM_17_20190907_AS.docx, Position: 3775-4411<br>I: Ok. So do you know how much balance was put every month?<br>R: No.<br>I: No? Who would get the balance done?<br>R: **He would get it done.**<br>I: Ok, your husband would get it done.<br>R; The one for Rs 10, right? He would do it.<br>I: He would do it. Ok. And would it ever happen that the phone has no balance? The balance would be zero.<br>R: We would get Rs 10 recharge.<br>I: Ok. And what after it gets over?<br>R: Then again.<br>I: Then how would you make a call?<br>R: I would only call when there was work.<br><br>Excerpt - Document: KL_WOM_18_20190910_AS.docx, Position: 9238-9430<br>I: You get a recharge? Ok. So the recharge, do you get it done, on your own?<br>R: **No, I tell him, the father of the kids. So he gets it done**<br><br>Excerpt - Document: KL_FAM_18_20190910_AJ.docx, Position: 5634-5900<br>I: When the recharge needs to be done then she does by her own or you do?<br>R: I only do…my younger brother stay with me since two years so he does the recharge. Sometimes I do recharge for her and sometimes brother does recharge for her.<br>I: Any time she goes for recharge?<br>R: **She does not go.**<br><br>Excerpt - Document: KL_WOM_09_20190901_AS.docx,<br>R: We do it from the house, or from the shop. For recharging the balance, we give money to the shopkeeper.<br>I: So for recharging the balance, do you go or your husband does it?<br>R: **My husband does it.**<br><br>Excerpt - Document: KL_WOM_16_20190907_SY.docx, Position: 50538-50778<br>I: Do you know how much balance is put every month?<br>R: I don't know.<br>I: You don't know.<br>R: Husband gets it done.<br>I: Ok, husband only gets it done.<br>R**: He doesn't tell me whether he got the balance added or not**<br><br>Excerpt - Document: KL_WOM_01_20190827_AJ.docx,<br>I: If you need to get it recharged, then who gets it done?<br>R: My husband does it.<br>I: Ok. Have you any idea about the amount he recharges it with, in a month?<br>R: **No, I don't know about this.**	- lower financial knowledge
Excerpt - Document: KL_FAM_24_20190912_AS.docx, Position: 3533-4770<br>I: So, do you recharge her phone or does she do it on her own?<br>R: **No, I do it. I am the earner, she is a lady, a housewife**. [Laughs]<br><br>Excerpt - Document: KL_WOM_29_20190914_AS.docx, Position: 1974-3127<br>I: How much did you recharge it for? You must have been recharging it with some balance.<br>R: Yes. The incoming was free.<br>I: It was unlimited?<br>R: Yes.<br>I: So what was the monthly amount you recharged it with?<br>R: One hundred and fifty.<br>I: Was it done by you or bhaiya (your husband)?<br>R: **My husband does it. I don't have any job**.	- Female domestic focus, male earning power

Dyads and quotations related to phone characteristics and functionality	Sub theme
Excerpt - Document: KL_WOM_18_20190910_AS.docx, Position: 9592-10842<br>R: **His responsibility is outside work, to earn money and my work is to take care of kids. If you think the kids need food, you get that for them as well.**<br><br>Excerpt - Document: KL_WOM_05_20190828_NP.docx,<br>I : Ok. Who does the job of putting money in the phone? It has to be recharged.<br>R : Husband.<br>I : Your husband does the recharge. For how much he does the recharge?<br>R : **That I don't know.**<br><br>Excerpt - Document: KL_WOM_07_20190829_SY.docx, Position: 7465-8226<br>I: Who recharges your phone?<br>**R: My husband does that. It is done in the market.**<br>**I: In the market? Is this the reason why your husband does it?**<br>**R: Yes.**	-lower autonomy to move freely outside the home
